# TRS-based PCR as a potential tool for inter-serovar discrimination of *Salmonella* Enteritidis, *S.* Typhimurium, *S.* Infantis, *S.* Virchow, *S.* Hadar, *S.* Newport and *S.* Anatum

**DOI:** 10.1007/s11033-014-3592-9

**Published:** 2014-07-26

**Authors:** Marta Majchrzak, Anna Krzyzanowska, Anna B. Kubiak, Arkadiusz Wojtasik, Tomasz Wolkowicz, Jolanta Szych, Pawel Parniewski

**Affiliations:** 1 Institute of Medical Biology PAS, 106 Lodowa Street, 93-232 Lodz, Poland; 2 Department of Bacteriology, National Institute of Public Health - National Institute of Hygiene, 24 Chocimska Street, 00-791 Warsaw, Poland

**Keywords:** *Salmonella enterica*, TRS-PCR, Genotyping, Serovars

## Abstract

*Salmonella enterica* subsp. *enterica* comprises a number of serovars, many of which pose an epidemiological threat to humans and are a worldwide cause of morbidity and mortality. Most reported food infection outbreaks involve the serovars *Salmonella* Enteritidis and *Salmonella* Typhimurium. Rapid identification to determine the primary sources of the bacterial contamination is important to the improvement of public health. In recent years, many DNA-based techniques have been applied to genotype *Salmonella*. Herein, we report the use of a manual TRS-PCR approach for the differentiation of the *Salmonella enterica* subspecies *enterica* serovars in a single-tube assay. One hundred seventy *Salmonella* strains were examined in this work. These consisted of serovars *S.* Enteritidis, *S.* Typhimurium, *S.* Infantis, *S.* Virchow, *S.* Hadar, *S.* Newport and *S.* Anatum. Five of the TRS-primers, N_6_(GTG)_4_, N_6_(CAC)_4_, N_6_(CGG)_4_, N_6_(CCG)_4_ and N_6_(CTG)_4_, perfectly distinguished the *S*. Enteritidis and *S*. Typhimurium serovars, and the N_6_(GTG)_4_ primer additionally grouped the other five frequently isolated serovars. In our opinion, the TRS-PCR methodology could be recommended for a quick and simple DNA-based test for inter-serovar discrimination of *Salmonella* strains.

## Introduction

*Salmonella enterica* subsp. *enterica* comprises a number of serovars, many of which pose epidemiological threats to humans worldwide. In the European Union, *S.* Enteritidis and *S.* Typhimurium are the most frequently reported serovars [1–3]. Human infections with serovar *S.* Enteritidis are predominately associated with the consumption of contaminated eggs and poultry meat, while *S.* Typhimurium cases are mostly associated with the consumption of contaminated pork, poultry and bovine meat [4]. Therefore, the European Commission has introduced the obligation to examine poultry for the appearance of *S.* Enteritidis and *S.* Typhimurium, according to which, during the entire period before expiration, there should be none of these serovars in a 25 g sample [5].

A wide range of other serovars, i.e., *S.* Infantis, *S.* Virchow, *S.* Hadar, *S.* Anatum, *S.* Newport, are commonly isolated in humans and also are of public health significance [1–3].

*Salmonella* isolates are currently phenotypically identified according to the White–Kauffmann–Le Minor scheme [6], even though this method is labor-intensive and expensive. In addition, several molecular typing methods have been developed and applied to distinguish *S*. *enterica* isolates. Pulsed-field gel electrophoresis (PFGE) is a “gold standard” among the subtyping methods used in *Salmonella* outbreak investigations [7]. Despite the undeniable advantage of employing the highly advanced molecular methods, the cost of equipment and need for skilled staff may exclude some methods from use in many countries that need them the most. That is why there is still a need for new methods that are simple, inexpensive and able to discriminate among *Salmonella* serotypes.

Together with our previous studies, this study shows the usefulness of the manual rep-PCR procedure based on the presence of trinucleotide repeat sequences (TRSs) dispersed throughout the bacterial genome. This method uses primers that are complementary to commonly occurring trinucleotide repeat DNA sequences. Previously, we evaluated the (CGG)_4_-based PCR for the discrimination of uropathogenic *Escherichia coli* [8], a (CAC)_4_-based PCR for the discrimination of *Mycobacterium gordonae* [9] and a (CCG)_4_-based PCR for the discrimination of *Mycobacterium kansasii* [10] and *Mycobacterium avium* [11]. In the present work, we examined a collection of 170 clinical *S. enterica* strains (Table 1). This collection consisted of the *S*. Enteritidis and *S*. Typhimurium serovars, which are the top two serovars isolated from humans in Poland and also serovars that are still of great clinical importance (*S.* Infantis, *S.* Virchow, *S.* Hadar, *S.* Anatum and *S.* Newport) [1]. The objective of the project was to implement a simple test that (i) is able to distinguish the *S.* Enteritidis and *S.* Typhimurium serovars and (ii) has the potential to discriminate among other serovars, such as *S.* Infantis, *S.* Virchow, *S.* Hadar, *S.* Anatum and *S.* Newport. This method could be used as a preliminary approach for *Salmonella* discrimination in order to reduce the cost of serotyping.

**Table 1:** *Salmonella enterica* subsp. *enterica* isolates used in this study

Serovar	Isolate no.	Antigenic formulae	Patient ID	Sex/age	Origin	Collection date [d.m.y]	Place of isolation ID^a^
Typhimurium	S003	1,4 [5],12:i:1,2	63	M/23y	stool	20.02.2009	1
	S005		1	M/30y	stool	20.02.2009	1
	S006		2	F/28y	stool	18.03.2009	1
	S013		2	F/28y	stool	31.03.2009	1
	S048		3	F/20 m	stool	27.08.2009	1
	S050		4	M/6y	stool	08.09.2009	1
	S061		5	M/25 m	stool	29.09.2009	1
	S062		6	M/25 m	stool	29.09.2009	1
	S064		7	F/19 m	stool	29.09.2009	1
	S077		8	F/12 m	stool	29.10.2009	1
	S111		?	?	stool	(04.04.2011)^b^	2
	S116		?	?	stool	(04.04.2011)	2
	S117		?	?	stool	(04.04.2011)	2
	S118		?	?	stool	(04.04.2011)	2
	S119		?	?	stool	(04.04.2011)	2
	S121		?	?	stool	(04.04.2011)	2
	S122		?	?	stool	(04.04.2011)	2
	S123		?	?	stool	(04.04.2011)	2
	S125		NA	NA	food	(10.04.2012)	NA
	S126		NA	NA	food	(10.04.2012)	3
	S127		66	M/58y	stool	22.02.2012	4
	S128		67	M/6	stool	18.10.2011	5
	S129		68	F/18 m	stool	04.09.2011	5
	S130		69	F/86	stool	01.09.2011	4
	S131		70	M/?	stool	(13.09.2011)	4
	S132		71	F/55	stool	01.08.2011	4
	S133		72	M/42 m	stool	06.08.2011	4
	S134		NA	NA	food	(23.03.2011)	NA
	S135		73	M/42 m	stool	15.11.2010	5
	S136		NA	NA	RIVM	NA	NA
	S137		NA	NA	food	(05.11.2010)	NA
	S138		74	F/26y	blood	15.09.2010	6
	S139		75	M/50y	blood	07.09.09	6
	S140		NA	NA	food	(13.10.10)	NA
	S141		NA	NA	food	(13.10.2010)	NA
	S142		NA	NA	food	(21.01.2010)	NA
	S143		NA	NA	RIVM	NA	NA
	S144		NA	NA	food	(17.11.2009)	NA
Infantis	S004	6,7,14:r:1,5	9	M/20 m	stool	20.02.2009	1
	S018		10	F/17 m	stool	23.04.2009	1
	S025		11	M/8 m	stool	14.05.2009	1
	S054		12	F/7 m	stool	08.09.2009	1
	S100		13	?	stool	(04.04.2011)	2
	S145		76	M/4 m	stool	01.02.2012	3
	S146		77	F/88y	urine	22.11.2010	6
	S147		NA	NA	RIVM	NA	NA
	S148		78	M/25y	stool	20.06.2010	3
	S149		NA	NA	RIVM	NA	NA
	S150		NA	NA	RIVM	NA	NA
	S151		79	F/?	stool	30.04.2008	1
	S152		80	K/51y	stool	06.06.2007	6
	S153		81	M/19y	stool	25.05.2007	6
	S154		82	M/3 m	stool	18.05.2007	6
	S155		83	F/8 m	stool	15.09.2006	7
	S156		?	?	stool	13.05.2006	8
	S157		?	?	?	(11.01.2006)	1
	S158		?	?	urine	(05.01.2006)	6
	S159		NA	NA	food	(30.11.2005)	NA
	S160		?	?	stool	(25.08.2005)	3
	S161		?	?	stool	05.03.2005	8
	S162		?	?	stool	(07.05.2004)	3
	S163		?	?	stool	(18.02.2004)	3
	S164		?	?	?	(05.01.2004)	3
Hadar	S002	6,8:z10:e,n,x	60	F/61y	stool	20.02.2009	1
	S012		61	F/17 m	stool	25.03.2009	1
	S104		?	?	stool	(04.04.2011)	2
	S185		NA	NA	RIVM	NA	NA
	S186		NA	NA	RIVM	NA	NA
	S187		NA	NA	RIVM	NA	NA
	S188		?	?	stool	22.10.2007	7
	S189		89	M/?	stool	28.07.2007	7
	S190		90	M/22y	stool	02.05.2007	6
	S191		91	M/19y	stool	24.05.2007	6
	S192		92	M/10y	stool	25.05.2007	6
	S193		93	F/18y	stool	05.06.2007	6
	S194		?	?	?	16.01.2007	6
	S195		?	?	?	16.01.2007	6
	S196		?	?	?	16.01.2007	6
	S197		NA	NA	food	(16.11.2006)	NA
	S198		NA	NA	food	(20.09.2006)	NA
	S199		NA	NA	food	(05.01.2006)	NA
	S200		?	?	blood	15.11.2005	3
	S201		?	?	stool	19.09.2005	3
	S202		NA	NA	food	(17.11.2005)	NA
	S203		NA	NA	food	(02.11.2005)	NA
Virchow	S027	6,7,14:r:1,2	14	F/16 m	stool	27.05.2009	1
	S034		15	F/9 m	stool	24.06.2009	1
	S035		15	F/9 m	stool	27.08.2009	1
	S036		16	M/7 m	stool	27.08.2009	1
	S107		17	?	stool	(04.04.2011)	2
	S114		18	F/4y	stool	13.09.2010	2
	S115		18	F/4y	stool	09.09.2010	2
	S120		19	?	stool	(04.04.2011)	2
	S165		84	M/18y	stool	20.10.2010	6
	S166		NA	NA	RIVM	NA	NA
	S167		?	?	?	(01.03.2010)	1
	S168		NA	NA	food	(07.01.2010)	NA
	S169		NA	NA	RIVM	NA	NA
	S170		85	M/?	blood	02.11.2009	6
	S171		?	?	stool	04.09.2009	8
	S172		?	?	stool	28.08.2009	8
	S173		?	?	stool	21.08.2009	8
	S174		86	F/?	stool	03.04.2009	7
	S175		86	F/?	stool	03.04.2009	7
	S176		86	F/?	stool	03.04.2009	7
	S177		NA	NA	RIVM	NA	NA
	S178		?	?	stool	12.11.2007	8
	S179		?	?	stool	07.09.2007	8
	S180		?	?	stool	24.08.2007	8
	S181		?	?	stool	25.07.2007	7
	S182		87	M/27y	stool	01.06.2007	4
	S183		88	M/?	tissue	(04.10.2006)	3
	S184		?	?	stool	(07.09.2006)	9
Enteritidis	S001	1,9,12:g,m:−	20	M/30 m	stool	29.01.2009	1
	S007		21	M/16 m	stool	18.03.2009	1
	S008		64	F/36 m	stool	18.03.2009	1
	S009		22	M/9y	stool	18.03.2009	1
	S014		25	M/28 m	stool	31.03.2009	1
	S015		26	M/5y	stool	16.04.2009	1
	S016		27	F/5y	stool	31.03.2009	1
	S017		25	M/29 m	stool	16.04.2009	1
	S021		25	M/29 m	stool	16.04.2009	1
	S022		28	F/11 m	stool	23.04.2009	1
	S023		29	F/16 m	stool	29.04.2009	1
	S024		30	F/10 m	stool	14.05.2009	1
	S028		31	M/76y	stool	14.05.2009	1
	S029		32	F/36 m	stool	14.05.2009	1
	S030		33	M/30 m	stool	10.06.2009	1
	S031		34	F/13 m	stool	10.06.2009	1
	S032		35	M/74y	stool	10.06.2009	1
	S033		36	F/4y	stool	10.06.2009	1
	S037		37	F/5y	stool	24.06.2009	1
	S039		38	F/15 m	stool	27.08.2009	1
	S040		39	M/27 m	stool	27.08.2009	1
	S041		40	M/4y	stool	27.08.2009	1
	S043		41	F/13 m	stool	27.08.2009	1
	S044		42	M/27 m	stool	27.08.2009	1
	S045		43	F/37 m	stool	27.08.2009	1
	S046		44	F/24 m	stool	27.08.2009	1
	S047		45	F/25 m	stool	27.08.2009	1
	S049		46	M/4y	stool	27.08.2009	1
	S052		47	M/21 m	stool	08.09.2009	1
	S053		48	F/22y	stool	08.09.2009	1
	S055		49	F/73y	stool	08.09.2009	1
	S056		50	M/24 m	stool	08.09.2009	1
	S063		51	M/22 m	stool	29.09.2009	1
	S065		52	M/26 m	stool	29.09.2009	1
	S066		53	F/17 m	stool	29.09.2009	1
	S067		54	F/87y	stool	29.09.2009	1
	S068		55	F/5y	stool	29.09.2009	1
	S069		56	M/9y	stool	13.10.2009	1
	S070		57	F/19 m	stool	13.10.2009	1
	S071		65	F/5y	stool	13.10.2009	1
	S073		58	F/11 m	stool	29.10.2009	1
Anatum	S026	3,{10}{15}{15,34}:e,h:1,6	59	M/4y	stool	27.05.2009	1
	S204		NA	NA	RIVM	NA	NA
	S205		96	F/71y	stool	14.05.2007	6
	S206		?	?	stool	(09.03.2005)	3
	S207		?	?	stool	(07.06.2003)	3
	S208		?	?	stool	(07.06.2003)	3
	S209		?	?	stool	21.05.2003	3
	S210		?	?	stool	21.05.2003	3
Newport	S083	6,8,20:e,h:1,2	62	M/6 m	stool	03.12.2009	1
	S211		NA	NA	RIVM	NA	NA
	S212		94	F/?	stool	17.07.2009	6
	S213		NA	NA	food	(20.08.2008)	NA
	S214		95	F/38y	stool	07.11.2007	6
	S215		?	?	?	(25.08.2005)	3
	S216		?	?	stool	(06.10.2003)	8
	S217		?	?	stool	27.02.2003	8

^a^The same number refers to the same region of Poland (voivodeship) but different hospital/diagnostic laboratory

^b^In brackets there is date of isolate receiving

? unknown, *NA*-not applicable, *F* Female, *M* Male, *y* years, *m* months

*RIVM*—strains obtained from The Netherlands National Institute for Public Health and the Environment

## Materials and methods

### Bacterial strains

All of the strains used in this study were collected from the SYNEVO Medical Laboratory (Lodz, Poland), National Institute of Public Health (Warsaw, Poland) and Institute of Genetics and Microbiology (University of Wroclaw, Poland) from June 2003 to April 2012 (Table 1). The RIVM strains were obtained from The Netherlands National Institute for Public Health and the Environment (Table 1). A total of 170 strains were isolated from humans and food samples with *Salmonella* infections in laboratories mentioned above and they were biochemically identified and serotyped by a slide agglutination test with specific O and H antisera, and classified according to the White–Kauffmann–Le Minor scheme [6]. We obtained clean, serologically characterized isolates that were used for further studies. The whole collection consisted of: 41 strains of *S.* Enteritidis, 38 strains of *S.* Typhimurium, 25 strains of *S.* Infantis, 28 strains of *S.* Virchow, 22 strains of *S.* Hadar, 8 strains of both *S.* Anatum and *S.* Newport.

### Bacterial growth and genomic DNA isolation

For further studies, after isolation of a single colony from SS Agar (*Salmonella Shigella* Agar), all of the isolates were grown in liquid LB broth at 37 °C overnight with an agitation speed of 120 RPM. The genomic DNA was isolated using a GenElute Bacterial Genomic DNA Kit (Sigma-Aldrich, St. Louis, MO). The purity and quantity of the DNA were determined spectrophotometrically at 260 nm (BioPhotometer, Eppendorf, Germany).

### TRS-PCR and fingerprint analysis

The primers were designed to conform to the 5’-N_6_(TRS)_4_-3′ scheme in which N represents G, A, T or C in a random manner. The TRS-PCR, electrophoresis, reproducibility assessments and bioinformatic analyses were performed as reported in previously published protocols [8–10], with the exception of the DNA template concentration. The TRS-PCRs were performed in a final volume of 50 µl using 10 ng of the isolated DNA, 1 U *Taq* polymerase (Invitrogen by Life Technologies, CA, USA), 1× polymerase buffer, 1.5 mM of MgCl_2_, 50 pmol of TRS-primer (each containing a single TRS motif), 0.2 mM of each deoxynucleoside triphosphate and 6 % DMSO. The PCR amplifications were accomplished using a T-3000 termocycler (Biometra, Goettingen, Germany) with an initial denaturation step (95 °C, 3 min) followed by 35 cycles of denaturation (95 °C, 1 min), annealing (variable temperatures —Table 2, 1 min), extension (72 °C, 2 min) and final extension step (72 °C, 8 min). The PCR products, 10 µl of 50 µl, were resolved by horizontal electrophoresis on 1.6 % agarose gel in a 1 × TAE buffer. Electrophoresis was performed at room temperature and 70 V (2.4 V/cm) until the dye (Bromophenol blue) migrated 6 cm from the wells (~2 h). Afterwards, gels were stained in an EtBr solution (0.5 µg/ml) for 10 min and destained in water for another 10 min. The images of the gels were captured under UV light using a FluorChem 8800 system with Alpha EaseFC v. 3.1.2 software (AlphaInnotech, CA, USA). The cluster analyses of the TRS-PCR and ERIC-PCR genomic profiles were carried out with BioNumerics software (Applied Maths, Belgium). The sizes of PCR products in each lane of the agarose gel were normalized with regard to the 100 bp DNA size marker (Fermentas, Thermo Scientific Waltham, MA, USA). The fingerprint similarity comparisons were calculated using a Pearson correlation (optimization 1 %, position tolerance 1 %) and grouping was done according to the UPGMA algorithm. The ERIC-PCR was performed as described elsewhere [8, 9, 12] except for the DNA concentration (~10 ng/µl). The reproducibility of TRS-PCR and ERIC-PCR was obtained by comparing the three separate fingerprints (from three different PCR runs) of one selected strain from each of the investigated serovars.

**Table 2:** Parameters of the TRS-PCR

TRS motif (direct/complementary)	Theoretical number of motifs (TRS) *n* ≥ 3^a^	Annealing temperatures of the TRS primers (°C)	Practical utility of the TRS primers^b^	Reproducibility of the band patterns (%)^c^
CGG/CCG	1,035	72	“+”	94.8/94.5
CTG/CAG	478	61		94.7/ND
GTG/CAC	294	55/61		95.7/96.6
ATG/CAT	267	44	“±”	ND
AAG/CTT	172	44		
GTC/GAC	140	61		
TTG/CAA	115	45		
TAT/ATA	203	<44	“−”	ND
TCC/GGA	42	61		
TAG/CTA	17	44		

^a^Based on in silico analysis of the genome of *Salmonella* Enteritidis str. P125109

^b^Based on PCR reactions, where “+” indicates fingerprints with good quality, “±” indicates fingerprints with poor quality, and “−” indicates no product

^c^The reproducibility of the TRS-PCR was obtained by comparing (Pearson correlation, UPGMA algorithm) the three separate fingerprints (from three different PCR runs) of one selected strain from investigated serovars; the numbers show the mean same strain similarity values

*ND* Not Determined

## Results

### *In silico* analysis

*In silico* analysis of the entire genome sequence data of *S.* Enteritidis (str. P125109, GenBank acc. no. AM933172) was conducted (Vector NTI 9.0.0.) to estimate the number of trinucleotide repeat tracts. This approach enabled us to predict the utility of the TRS-containing primers. There are 64 possible combinations of trinucleotide repeats. However, after eliminating four mononucleotide repetitions as well as taking into account the fact that each of the motifs can be written as three equivalent frames (i.e., CTG = TGC = GCT), it appears that only 20 motifs are sufficient for planning a complete set of primers for the TRS-PCR test. The theoretical calculations yielded a number of TRS motifs scattered on both strands and not the number of possible amplicons that may be generated by PCR (Table 2). Therefore, we decided to implement the TRS-based PCR separately for each of the 20 primers.

### Reference method

To select a rep-PCR-based test as the reference method, we performed three manual rep-PCRs, as follows: REP-PCR (primers REP-2I and REP-1R), BOX-PCR (primer BOX-A1R) and ERIC-PCR (primers ERIC-1R and ERIC-2). These typing methods were formerly used for gram-negative enterobacterial strain differentiation [12–16] and, as well as TRS-PCR, rely on an amplification of genomic DNA fragments using sets of primers complementary to the short repetitive sequences. Among REP-, BOX- and ERIC-PCR methods, only ERIC-PCR produced fingerprints with good quality and resolution (data not shown); therefore, this method was chosen as the rep-PCR reference method for typing the 170 isolates of *S*. *enterica*.

### TRS-based PCR: preliminary analysis

Preliminary tests were conducted on a collection of 32 strains from the seven investigated serovars (10 strains of *S.* Enteritidis, 10 strains of *S.* Typhimurium and three strains from each of the remaining serovars: *S.* Virchow, *S.* Infantis, *S.* Newport, *S.* Anatum). In these studies, 14 of the 20 primers with TRS motifs produced fingerprints. Four of the primers, containing the motifs TCC, AGG, TAG and TAC, produced no products, as was expected from our in silico analysis (low theoretical number of TRS motifs, Table 2). In the case of the primers harboring the TAT and ATA motifs, the annealing temperature (below 44 °C) probably did not allow the amplification of any product. Eight primers, containing the motifs GTC, GAC, TTG, AAC, AAG, TTC, ATG and ATC, produced poor-quality profiles (data not shown). Six primers, containing the motifs CAC, CGG, CCG, CTG, CAG and GTG, produced complex fingerprints with good resolution and discrimination potential. However, only five of these primers (all except CAG) fulfilled the first of our assumptions, that is, distinguishing the *S.* Enteritidis and *S.* Typhimurium serovars.

### TRS-based PCR: inter-serovar discrimination

**Fig. 1:**
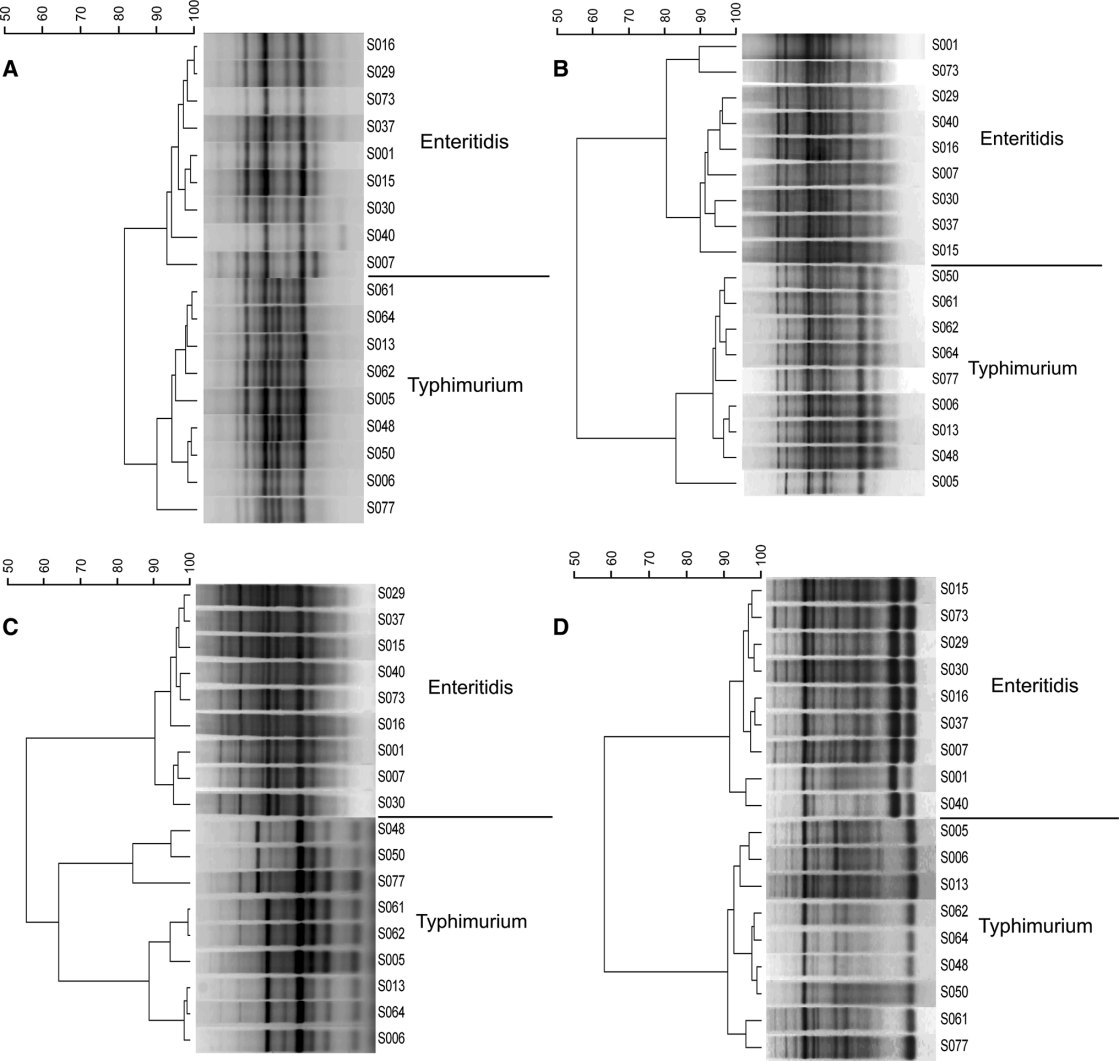
**a** N_6_(CAC)_4_-based, **b** N_6_(CGG)_4_-based, **c** N_6_(CCG)_4_-based and **d** N_6_(CTG)_4_-based band pattern comparison of *Salmonella* Enteritidis and *Salmonella* Typhimurium strains. The similarities between fingerprints were calculated using the Pearson correlation (optimization 1.00 %, position tolerance 1.00 %) and the fingerprints were grouped by use of the UPGMA algorithm

The TRS-based band pattern analyses employing N_6_(CAC)_4_, N_6_(CGG)_4_, N_6_(CCG)_4_ and N_6_(CTG)_4_ primers for the *S.* Enteritidis and *S.* Typhimurium strains are shown in Fig. 1a, b, c and d, respectively. Isolates of the same serovar clustered together and were represented by similar fingerprints. Moreover, PCR genotyping with the N_6_(GTG)_4_ primer generated highly uniform fingerprints for all seven serotypes, therefore, this primer was used for analysis of the whole 170 *Salmonella enterica* subsp. *enterica* strain collection.

**Fig. 2:**
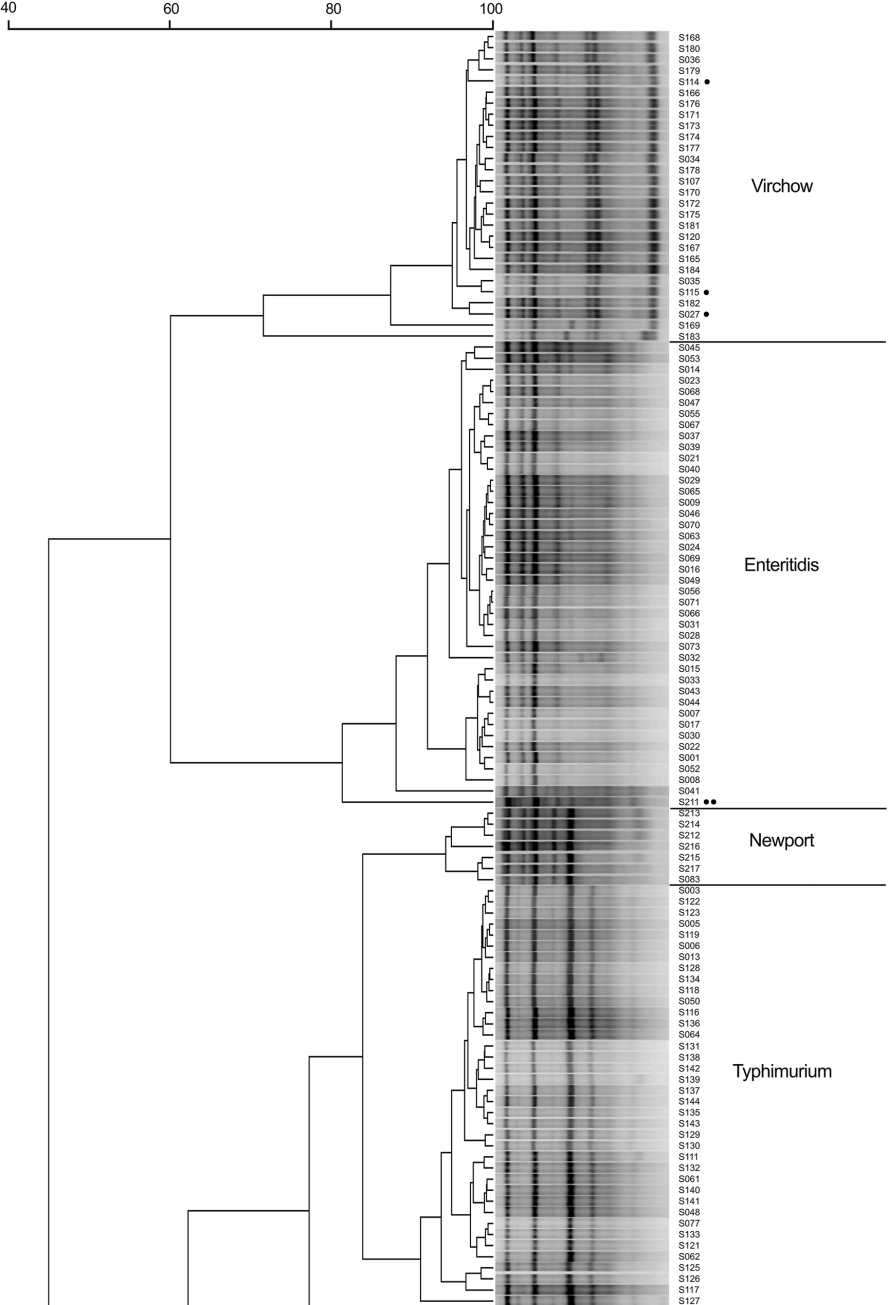

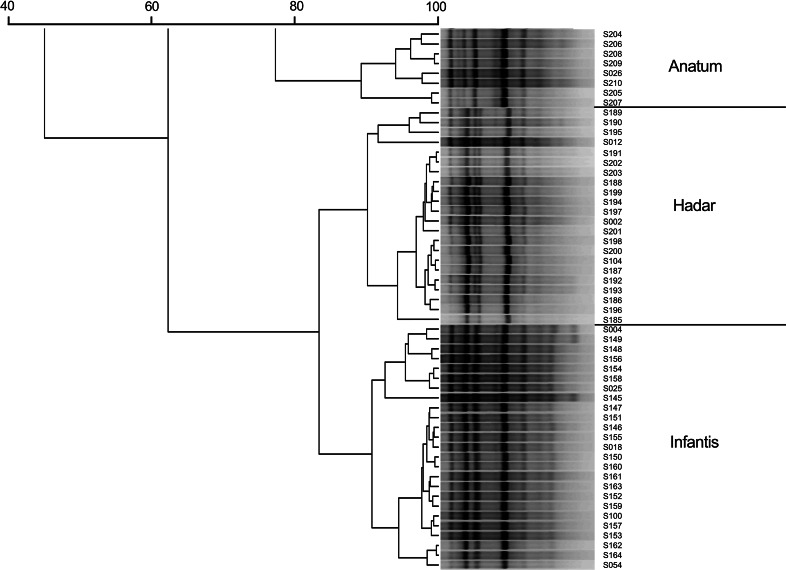
N_6_(GTG)_4_-based fingerprint similarity comparison of 170 *Salmonella enterica* subsp. *enterica* strains. The similarities between fingerprints were calculated using the Pearson correlation (optimization 1.00 %, position tolerance 1.00 %) and the fingerprints were grouped by use of the UPGMA algorithm. •—strains originally classified as *S*. Infantis; ••—strain S211 re-identified as *S*. Bardo

With use of (GTG)_4_-based PCR it was possible to classify *Salmonella* isolates into genetically related clusters that were, for the most part, homogeneous for serotype (Fig. 2). However, there were some inaccuracies with strain S211, described as *S.* Newport (marked with a double dot, Fig. 2). Further investigations showed that this strain is in fact *S*. Bardo (I 8:e,h:1,2), which is very similar to *S.* Newport (I 6,8:e.h:1,2). Classical serotyping by slide agglutination test with specific O and H antisera may be susceptible to colonial form variations that may occur with the expression of the O:6 antigen [17]. Hendriksen et al. [18] conceded, for needs of WHO Global Salm-Surv EQAS, that both identifications could be treated as correct. Although, phenotypically such serotypes could converge on each other, our results suggest that genotypically they remain different. Interestingly, an additional serotype analysis performed with a Premi^®^Test *Salmonella* microarray (check-points, Netherlands, data not shown) has confirmed the wrong classification of this strain as *S.* Newport. Notably, the (GTG)_4_-based PCR analysis was also capable of revealing errors in laboratory documentation. Strains S027, S114 and S115 were originally classified as *S*. Infantis (strains marked with a single dot, Fig. 2). Their (GTG)_4_-based fingerprints were visibly different from the profiles of the serotypes to which they were assigned. In our analyses, these strains grouped with *S*. Virchow, which was confirmed by serotyping re-analysis.

(GTG)_4_-based PCR clustering analysis showed that similarities of strains within serovars *S.* Enteritidis, *S.* Typhimurium, *S.* Virchow, *S.* Infantis, *S.* Hadar, *S.* Newport and *S.* Anatum were 88, 91.1, 71.6, 90.7, 90.1, 94.2 and 89.2 %, respectively (Table 3, bold values). From these values, serovar *S*. Virchow seemed to be more variable. However, Fig. 2 shows that although two strains—S169 and S183—differed slightly from fingerprints of the other strains in the respective group, they still remained within the group. Cluster-to-cluster analysis demonstrated that similarities among serovar clusters were lower than the pattern similarity for all of the strains in a given cluster (Table 3).

**Table 3:** Inter- and intra-cluster similarities [%] based on GTG-PCR band patterns of 7 *Salmonella* serovars

	Virchow	Enteritidis	Newport^a^	Typhimurium	Anatum	Hadar	Infantis
Virchow	**71.6**	60.4	35.9	42.4	31.5	15.2	29.8
Enteritidis	60.4	**88.0**	67.8	61.0	50.3	35.6	55.8
Newport^a^	35.9	67.8	**94.2**	83.9	76.5	65.7	68.9
Typhimurium	42.4	61.0	83.9	**91.1**	77.3	57.5	60.3
Anatum	31.5	50.3	76.5	77.3	**89.2**	63.5	81.5
Hadar	15.2	35.6	65.7	57.5	63.5	**90.1**	83.4
Infantis	29.8	55.8	68.9	60.3	81.5	83.4	**90.7**

^a^Without strain S211; values in bold indicate intra-serovar similarities

### Reproducibility of TRS-PCR and ERIC-PCR

The reproducibility of TRS-PCR was calculated for the three chosen strains representing each serovar according to previously published protocols [8, 9, 11]. In the current reproducibility analysis, the mean same-strain similarity values were also high (Table 2). The ERIC-PCR exhibited significantly lower reproducibility (77 %) and was not able to cluster all of the strains properly (data not shown).

Taking all the above into consideration, a (GTG)_4_-based PCR was useful for effective, reproducible, inter-serovar discrimination of this *Salmonella* collection.

## Discussion

The use of rep-PCR-based genotyping for *Salmonella enterica* using the (GTG)_5_ primer has been published previously. Rasschaert et al. [16] concluded that the composite dataset for ERIC and the (GTG)_5_ primers provided serotype discrimination and suggested this rep-PCR be used to limit the number of strains that had to be serotyped. However, the authors emphasized that the reproducibility of the tests was lower if the isolates were analyzed during different PCR runs, and that there were two strains of *S.* Enteritidis that fell out of the main cluster of this serovar. Because we aimed to identify an easy, rapid and reproducible method for the differentiation of *Salmonella* isolates, the use of a single primer was more desirable than the composite analysis. We designed a set of TRS primers according to a 5′-N_6_(TRS)_4_-3′ scheme. In our case, the additional N_6_-tail at the 5′ end allows better anchoring to the various TRS-loci of the genomic template. Therefore, in our opinion, the use of a single primer—N_6_(GTG)_4_—was sufficient to obtain reproducible and satisfactory results.

Formerly, the (GTG)_5_-PCR technique was found to be a rapid and simple tool to reproducibly discriminate among a wide range of *Lactobacillus* species [19]. Also, this method was successfully applied in the typing of fecal and environmental *E. coli* isolates in comparison with other rep-PCR methods, including ERIC-PCR, REP-PCR and BOX-PCR [20]. In other studies, methods using the (GTG)_5_ primer were evaluated for the identification of *Streptococcus mutans*, *Bacillus* spp. and *Klebsiella* isolates [21–23]. However, these studies lacked reproducibility analyses, and there were some inaccuracies in the grouping of the bacterial isolates.

In our collection, there were no *S*. Dublin strains, which are closely related to *S*. Enteritidis and 4,5,12:i:—strains representing a monophasic variant of *S*. Typhimurium. Thus, we could not verify if our test would be able to distinguish these serovars properly. Such analyses are in progress but still require some further investigations. The range of serovars examined in our studies was limited; therefore, it would be desirable to investigate a more diverse population of *Salmonella enterica* strains in the future. Herein, we report that the N_6_(GTG)_4_-PCR methodology can be used for rapid and easy single-tube DNA-based assays for the discrimination of seven *S. enterica* subsp. *enterica* serovars. The determination of TRS fingerprints for unknown *Salmonella* strains could serve as a useful predictor for their serovar affinity. Although conventional serotyping should still be performed, a rapid screen with TRS-based PCR may greatly reduce the number of antisera used for determination of *Salmonella* serovars and may help prioritize further investigation of *Salmonella* strains. It seems to be useful not only for examination of strains isolated from humans but also as a pilot survey of poultry, according to Commission Regulation No 1086/2011 [5].
